# Description of Atrial Fibrillation in a Cardiology Department in Mahajanga, Madagascar

**DOI:** 10.7759/cureus.82282

**Published:** 2025-04-15

**Authors:** Rova Malala Fandresena Randrianarisoa, Mirantosoa Fabiola Ravelonjatovo, Gerda Menzato, Narindrarimanana Avisoa Randriamihangy, Fidiarivony Ralison

**Affiliations:** 1 Internal Medicine, University Hospital Joseph Raseta Befelatanana, Antananarivo, MDG; 2 Cardiology, Mahavoky Atsimo University Hospital, Mahajanga, MDG; 3 Faculty of Medicine, University of Mahajanga, Mahajanga, MDG; 4 Internal Medicine, Mahavoky Atsimo University Hospital, Mahajanga, MDG

**Keywords:** atrial fibrillation, cardiovascular risk factors, diagnostic, madagascar, prevalence, treatment

## Abstract

Introduction

Despite the increasing development of research on atrial fibrillation (AF), epidemiologic data remain limited in certain regions, particularly Madagascar. Madagascar is a country with limited resources and data on the cardiovascular health of the population. Our objectives were to report the prevalence of AF and to describe patient characteristics.

Materials and methods

This was a retrospective descriptive study that included 103 patients with AF during their hospitalization at the cardiology department of the Mahavoky Atsimo University Hospital in Mahajanga between January 2015 and July 2023.

Results

The prevalence of AF was 5.27%. The mean age of the patients was 59.53 years (±17.88), and the sex ratio was 1.10. All patients were symptomatic, and dyspnea (53.40%; n = 55) was the main symptom. Signs of heart failure (HF) were observed in 70.87% of cases (n = 73). The majority of patients (87.38%; n = 90) had a CHA_2_DS_2_-VASc (congestive HF, hypertension, age ≥75 years, diabetes mellitus, previous stroke, vascular disease, age 65-74 years, sex category) score ≥ 2, and of these, 10 patients were not anticoagulated. Vitamin K antagonists and direct oral anticoagulants were prescribed in 71.84% (n = 74) and 9.71% (n = 10) of cases, respectively. Rate control was the most commonly used therapeutic strategy. Spontaneous return to sinus rhythm occurred in 14 patients (13.59%). Twelve patients (11.65%) had a stroke, and one patient (0.97%) died during a mean hospital stay of 6.92 days (±4.7).

Conclusions

This study provides an overview of the situation in Madagascar. Management is far from meeting current recommendations for good practice, which is a major challenge.

## Introduction

Atrial fibrillation (AF) is a supraventricular arrhythmia characterized by rapid, uncoordinated atrial activity. It is associated with significant morbidity and mortality due to a fivefold increased risk of stroke, a threefold increased risk of heart failure (HF), and a nearly twofold increased risk of all-cause mortality [1]. It is also a major risk factor for dementia and cognitive decline [2]. This disease is closely related to atherosclerosis, whether coronary artery disease or cerebrovascular disease [3]. AF is the most common arrhythmia. Worldwide prevalence has tripled in the last 50 years and is expected to reach 52 million by 2021 [4]. This trend is expected to continue in the coming decades as the population ages, comorbidities increase, and diagnostic tools improve. In the United States (US), at least three to six million people have AF, and this number is expected to increase to six to 16 million by 2050 [5]. In Europe, the prevalence was estimated to be nine million people over the age of 55 in 2010 and is expected to reach 14 million by 2060 [5].

In Sub-Saharan Africa (SSA), health systems face the simultaneous challenges of communicable diseases and the rise of noncommunicable diseases, particularly cardiovascular disease (CVD) [6]. AF is a major public health problem in the African population, contributing to a significant increase in the burden of CVD between 1990 and 2010 [7]. By 2050, the prevalence of AF is projected to be higher in SSA than in any other region of the world [6]. However, available data on the epidemiology, treatment patterns, and impact of AF remain limited in some regions, such as Madagascar. The characteristics of Malagasy patients may differ from those reported in the literature due to the younger population and limited diagnostic and treatment resources. Conducting this study would allow us to establish the cardiovascular health profile of this specific population and add new information on the distribution of risk factors in the African literature. The present study aimed to report the prevalence of AF and to describe the characteristics of patients seen in a cardiology department in Madagascar.

## Materials and methods

Study features

This was a retrospective descriptive study of patients admitted to the cardiology department of the Mahavoky Atsimo University Hospital in Mahajanga. Mahajanga is located in the northwest of Madagascar and is the capital of the Boeny region. Opened in 2013, the cardiology unit currently has 16 conventional hospital beds and receives 241 inpatients per year.

Study population

The study involved all patients over the age of 18 who were hospitalized in the department for a period of eight years and seven months, from January 2015 to July 2023.

Inclusion and exclusion criteria

Patients presenting with AF during hospitalization, regardless of the reason for admission and associated diagnoses, were included in the study. AF was defined by the absence of distinct P waves and the presence of irregular R-R intervals on the 12-lead electrocardiogram (ECG) [8]. Patients receiving anticoagulant therapy for a condition other than AF and those with congenital heart disease, because of their different AF pathophysiology, were excluded. We also excluded patients with incomplete medical records that did not include sex or age.

Study parameters

Data were collected from patients' medical records. Variables studied included demographics, medical history, self-reported toxic use, clinical presentation, transthoracic echocardiography (TTE) and ECG characteristics, treatment, and occurrence of stroke and death. Several series of ECGs during hospitalization were checked.

Left-sided HF was evoked by dyspnea and crackles on lung auscultation. Dyspnea was staged according to the New York Heart Association (NYHA) classification: stage I (no limitation of physical activity), stage II (dyspnea on exertion), stage III (dyspnea on moderate and usual exertion), and stage IV (dyspnea at rest). Right-sided HF was induced by evidence of right-sided overload. HF was classified into three types according to the left ventricular ejection fraction (LVEF) assessed on TTE: reduced (≤ 40%), moderately reduced (41-49%), and preserved (≥ 50%) [9].

AF is considered tachycardic if the resting heart rate (HR) is ≥ 110 bpm and bradycardic if the HR is < 110 bpm [8]. To assess the risk of thromboembolism, the CHA_2_DS_2_-VASc (congestive HF, hypertension, age ≥75 years, diabetes mellitus, previous stroke, vascular disease, age 65-74 years, sex category) score was used, with points assigned for HF, arterial hypertension (AH), age ≥ 75 years (2 points), diabetes mellitus, history of stroke (2 points), and vascular disease, age 65-74 years, and female sex [8].

Statistical analysis

Qualitative variables were presented as proportions. Quantitative variables were presented as means. Data were entered using Microsoft Excel version 2016 (Microsoft Corporation, Redmond, US) and then analyzed using Epi Info version 7 (Centers for Disease Control and Prevention, Atlanta, US).

Ethical concerns

The study complied with the most recent and relevant ethical guidelines. The study was approved by the Research Ethics Committee of the University of Mahajanga. Patient anonymity and confidentiality were maintained.

## Results

**Figure 1:**
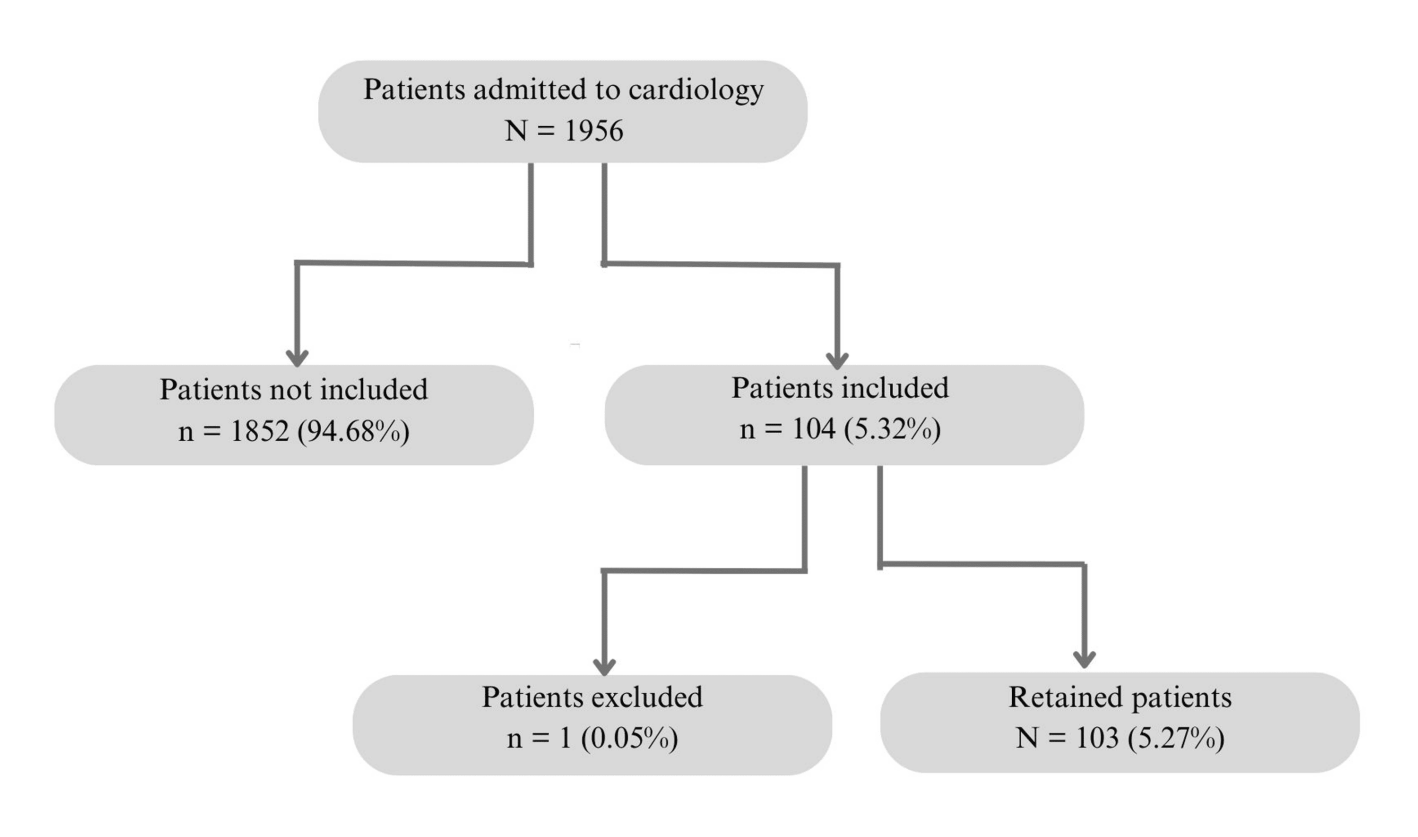
Patient selection flow chart

Figure 1 illustrates the patient selection flowchart. A total of 103 patients were included in this study, giving a hospital prevalence of 5.27%. The sex ratio was 1.10 with 54 men and 49 women. The mean age of the patients was 59.53 years (±17.88), with extremes of 19 and 90 years, and the age group ≥ 60 years (56.31%; n = 58) was the most represented. Nineteen patients (18.45%) were chronic alcoholics, and 17 (16.50%) were active smokers. None of the patients had a prosthetic valve.

**Figure 2:**
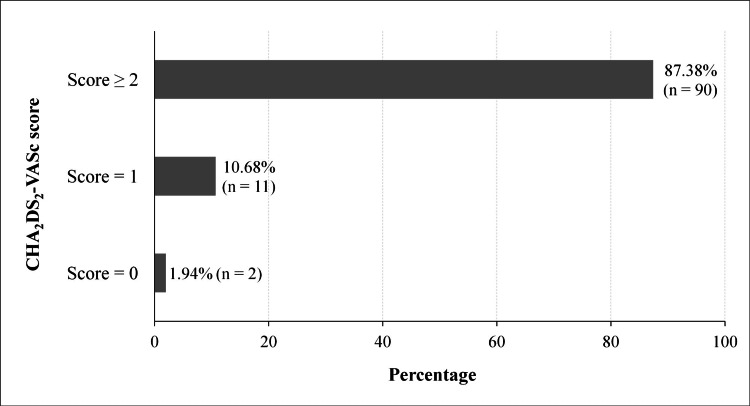
Patient distribution by CHA2DS2-VASc score CHA_2_DS_2_-VASc: congestive heart failure, hypertension, age ≥75 years, diabetes mellitus, previous stroke, vascular disease, age 65-74 years, sex category

Table 1 shows the clinical characteristics of the patients. Dyspnea was the most common symptom and was observed in 53.40% of patients (n = 55). Dyspnea was NYHA stage III in 50.90% (n = 28) and NYHA stage IV in 47.27% (n = 26). On admission, the mean HR was 87.67 bpm (±20.72), with extremes of 40 and 150 bpm. AF was tachycardic in 15.53% (n = 16) and slowed in 84.47% (n = 87). Seventy-three patients (70.87%) showed signs of HF. Figure 2 shows the distribution of patients by CHA_2_DS_2_-VASc score. The median CHA_2_DS_2_-VASc score was 3.

**Table 1:** Clinical characteristics of patients

Clinical characteristics		Number (N = 103)	Percentage (%)
Medical history	Arterial hypertension	55	53.40
	Heart failure	29	28.16
	Diabetes mellitus	13	12.62
	Stroke	9	8.74
	Known atrial fibrillation	7	6.80
	Mitral valve disease	2	1.94
	Hyperthyroidism	2	1.94
	Ischemic heart disease	1	0.97
Symptoms	Dyspnea	55	53.40
	Palpitations	21	20.39
	Focal neurological signs	11	10.68
	Lower limb edema	6	5.83
	Vertigo	4	3.88
	Asthenia	3	2.91
	Syncope	2	1.94
	Chest pain	1	0.97
Types of heart failure	Biventricular heart failure	34	33.01
	Right-sided heart failure	23	22.33
	Left-sided heart failure	16	15.53

TTE was performed in 77 patients. Table 2 shows the main morphological and functional abnormalities observed on TTE. Twelve patients (15.58%) had mitral valve disease, including eight with mitral regurgitation and four with mitral stenosis. The mean LVEF was 51.69% (±12.61), with extremes of 24% and 78%.

**Table 2:** Echocardiographic abnormalities LVEF: left ventricular ejection fraction

Observed anomalies		Number (n = 77)	Percentage (%)
Morphological abnormalities	Left atrial dilatation	36	46.75
	Left ventricular hypertrophy	30	38.96
	Mitral valve disease	12	15.58
	Intracardiac thrombus	1	1.30
	Kinetic dysfunction	54	70.13
	Diastolic dysfunction	10	12.99
LVEF	Preserved (≥ 50%)	40	51.95
	Moderately reduced (41-49%)	19	24.67
	Reduced (≤ 40%)	18	23.38

Table 3 shows the main AF therapeutic classes used in the patients. In the majority of cases, rate control was achieved with a mixture of drugs, alone or in combination. Amiodarone per os was used at low doses as a precautionary measure for cardioversion. No patient was treated with pharmacological or electrical cardioversion. Of the 90 patients with a CHA_2_DS_2_-VASc score ≥ 2, 10 patients (11.11%) were not anticoagulated due to financial concerns.

**Table 3:** Specific treatment of atrial fibrillation

Treatment		Number (N = 103)	Percentage (%)
Anticoagulation	Vitamin K antagonists	74	71.84
	Direct oral anticoagulants	10	9.71
Rate control	Beta-blockers	45	43.69
	Digitalis	35	33.98
	Amiodarone	24	23.30

Table 4 shows the evolution of the patients during hospitalization. The mean hospital stay was 6.92 days (±4.7). Twelve patients (11.95%) had thromboembolic complications, all in the form of stroke, one of whom was not anticoagulated. One patient died of a stroke confirmed by imaging.

**Table 4:** Patient outcome

Evolution		Number (N = 103)	Percentage (%)
Restoration of sinus rhythm	Yes	14	13.59
	No	89	86.41
Thromboembolism complication	Yes	12	11.65
	No	91	88.35
Patient discharge status	Alive	102	99.03
	Deceased	1	0.97

## Discussion

The distribution of AF varies according to socioeconomic status, ethnicity, genetic factors, and health resources [4,5]. This study focuses on the Malagasy population, a Black population with limited resources and epidemiologic data on cardiovascular health.

Prevalence of AF

The hospital prevalence of AF was 5.27%. Similar results have been reported in the Malagasy literature. In Antananarivo, the capital of Madagascar, Rakotonaivo et al. reported a prevalence of 5.4% at the Joseph Raseta Befelatanana Hospital in 2020 [10]. In Toamasina, the second largest city northeast of the capital, Rakoto Sedson et al. reported a prevalence of 4.9% at the Morafeno Hospital in 2023 [11]. However, it is very likely that AF would be underdiagnosed in our study because only symptomatic forms were hospitalized, which may underestimate the true prevalence of AF.

In the African literature, the prevalence of AF is highly variable. According to a meta-analysis by Noubiap et al. in 2019, the prevalence in patients admitted to cardiology departments varied from 2.7% to 29.8%. In the general population, the rate varied from 0.3% to 4.3% [1]. These variations may be due to differences in sample size and methodology. These data suggest that the prevalence of AF in SSA may be higher than previously estimated and underscore the importance of conducting more community-based epidemiologic studies with adequate representativeness. Simple methods such as mobile ECGs can be performed in the community.

Patient demographics

The mean age of patients in our study was 59.53 years. Rakotonaivo et al. [10] and Rakoto Sedon et al. [11] reported a mean age of 60.38 and 61.9 years, respectively. Other African studies reported a mean age of 63 years in Senegal in 2022 [12] and 59 years in Mali in 2024 [13]. In developed countries, the incidence and prevalence of AF are highest in the 70-74 age group, with a peak over 95 years of age [4]. Patients in SSA are therefore generally younger. This could be explained by the relatively young age of the population combined with difficulties in access to care. Some authors attribute this discrepancy to the high incidence of rheumatic heart disease, a heart disease of young subjects and often the cause of AF in SSA (nearly 22% of African patients with AF) [6,11,12].

We observed a male predominance with a sex ratio of 1.10, confirming the results of previous studies in Madagascar [10,11]. This gender disparity could be the result of a complex interaction between biological and psychosocial factors, such as hormonal protection of young women against CVD. It may also reflect population trends in health-seeking behavior. On the other hand, some studies have shown a higher incidence of AF in women compared to men [12-14]. However, an older Framingham study found no significant difference in the incidence of AF between the sexes [15].

Risk factors and clinical aspects

In our study, more than half of the patients (53.4%) were hypertensive. AH is the most important risk factor in patients with AF, with a frequency ranging from 30% to 75% [1,16]. AH contributes to the development of AF and increases the risk of cardiovascular complications. It often coexists with other modifiable and nonmodifiable risk factors and contributes to AF recurrence and readmission [17]. AH is undertreated in SSA, with many patients receiving no treatment or erratic and inconsistent treatment [6]. Optimal blood pressure control is critical in the management of AF and must be part of a comprehensive approach to risk factors.

AF can be asymptomatic, although 90% of patients report symptoms [18]. Symptoms associated with AF episodes are variable and have a significant impact on patients' quality of life. However, the presence or absence of symptoms does not appear to correlate with embolic complications or mortality [8]. In our study, all patients were symptomatic. Dyspnea (53.40%) and palpitations (20.39%) were the main symptoms, which are consistent with the results of several studies [10,14,15]. In fact, dyspnea and palpitations are the most common reasons for the detection of this arrhythmia. The predominance of NYHA stage III (50.90%) and IV (47.27%) dyspnea in the study reflects the late presentation of patients.

About 70.87% of patients had evidence of HF. HF is frequently associated with AF, and their coexistence significantly increases morbidity and mortality. Approximately two-thirds of AF patients have HF of varying severity. It can be either a risk factor or a complication of AF [5,16,19]. In a Malagasy study reported by Randriamihangy et al. in 2020, AF was ranked third among the factors leading to HF decompensation in patients seen at the Joseph Raseta Befelatanana Hospital [20]. Early detection and appropriate management of AF in patients with HF is crucial, as is appropriate management of HF in patients with AF. Furthermore, the high frequency of HF with preserved LVEF (51.95%) indicates a less severe form of HF, which could be attributed to the relatively young age of our patients.

Regarding the type of AF, studies have shown that the permanent form predominates in SSA, in contrast to the US and Europe [1,12]. This may be due to a lack of recording of first episodes of AF, limited availability of ECG equipment in some healthcare facilities, and less frequent use of rhythm control therapies. In our study, the classification of AF according to chronology was complicated by the absence of previous ECGs and the fact that patients were seen only at a symptomatic stage. This limitation has a significant impact on therapeutic decisions, including rhythm or rate control. Nevertheless, we found that 6.80% of patients had a known history of AF. This low frequency is explained by the absence of previous ECGs and population screening obstacles.

Thromboembolism risk and anticoagulation

In our study, 87.38% of patients had a CHA_2_DS_2_-VASc score ≥ 2. Vitamin K antagonists (VKAs) and direct oral anticoagulants (DOACs) were used in 71.84% and 9.71% of cases, respectively. In the meta-analysis by Noubiap et al., at least 80% of patients were eligible for anticoagulant treatment, with VKAs being the most commonly used [1]. In the absence of specific recommendations in SSA, practitioners often refer to the guidelines of European and American societies. According to these guidelines, DOACs are preferred to VKAs due to their lower bleeding risk and limited drug-drug interactions [8,21]. Our results show a significant deviation from good practice guidelines for the management of AF, especially as difficulties remain in the regularity of anticoagulant therapy. The high cost of DOACs makes them unaffordable for many patients in SSA. In addition, 11.11% of our patients with a CHA_2_DS_2_-VASc score ≥ 2 were not anticoagulated for financial reasons. The majority of these patients had low incomes and would need to pay for their own healthcare due to the lack of universal health coverage [22,23].

AF was complicated by stroke in 11.95% of cases. Available studies report that 10-15% of AF patients develop stroke, with rates ranging from 2.4% to 33.8% [1]. Although specific data on African populations are limited, stroke appears to occur at an earlier age in SSA than in high-income countries. In addition, the clinical profile of stroke in this region appears to have distinct features [6,24]. Our findings point to a critical gap in our healthcare system. This can be prevented by timely anticoagulation and appropriate management of risk factors such as HA, diabetes, and rheumatic diseases. However, our stroke prevalence may be underestimated because only symptomatic forms of AF were hospitalized and a significant number of paroxysmal AF cases may have been missed by 12-lead ECG. Unfortunately, Holter ECG and telemetry are not available in Mahajanga.

Rate and rhythm control

Our patients were treated with a mixture of beta-blockers, digitalis, and amiodarone, alone or in combination, to control HR. No patient received cardioversion therapy due to a lack of treatment and monitoring equipment. According to the literature, rate control is the most commonly used therapeutic strategy in SSA [6,12]. It is used in approximately 65% to 95% of AF patients, compared to 6% to 36% for rhythm control [1]. This represents a major obstacle in the management of AF due to the inaccessibility of advanced therapies for rhythm control and the lack of TTE to guide treatment in some patients [23]. This therapeutic approach contrasts with that observed in developed countries. According to the Registry on Cardiac Rhythm Disorders Assessing the Control of Atrial Fibrillation (RECORDAF) observational study, rhythm control was performed in 55% of patients diagnosed in Europe, America, and Asia [25].

Spontaneous return to sinus rhythm was observed in 14 patients (13.59%), suggesting paroxysmal AF. In the study by Diop et al. in Senegal, restoration of sinus rhythm was reported in five patients (2.97%) [12]. Similarly, in the study by Sanogo et al. in Mali, only one patient (1.96%) recovered sinus rhythm [13]. The low percentage of sinus rhythm restoration observed in the study could be attributed to the fact that rhythm control was the least frequently used therapeutic strategy in the management of AF. Therefore, it may reflect the predominance of permanent AF.

AF mortality

AF is associated with an increased risk of death. According to the Global Burden of Disease 2021 report, it was responsible for more than 340,000 deaths worldwide, with higher mortality observed in low- and middle-income countries [4]. HF has been identified as the leading cause of death in patients with AF [26]. In SSA, stroke is the leading cardiovascular cause of death [7]. In our study, one patient (0.97%) died of stroke during a mean hospital stay of 6.92 days (±4.7). In the study by Rakoto Sedson et al., the mortality rate was 10% [11]. In Mali, 15.7% of patients died during a mean hospital stay of seven days (±3) [13]. Our mortality rate is therefore lower than these data. This may reflect our short follow-up.

Study strengths and limits

Our study has several limitations. The hospital prevalence of AF may be underestimated because of the small number of patients included, the fact that only symptomatic forms were hospitalized, and the significant number of cases of AF that may not have been detected by 12-lead ECG. As a reminder, the 12-lead ECG cannot detect paroxysmal AF. Chronological classification of AF was not performed, which has a significant impact on therapeutic decisions. The etiologies of AF could not be assessed due to the absence of cardiac biomarkers, thyroid function tests, and TTE in some patients. In future research, it would be interesting to assess the prevalence of subclinical AF and to follow patients with AF over a longer period of time.

Although this study does not reflect the exact prevalence of AF in the general population, it provides valuable insights into the reality of this arrhythmia in Madagascar. The management of AF does not appear to meet current good practice recommendations, which is a major challenge. The study highlights the need to improve diagnostic tools for AF detection, such as ECG Holter and telemetry. It also highlights the importance of optimizing therapeutic strategies for stroke prevention by including DOACs in essential drug lists and facilitating access to generic alternatives to DOACs. Finally, it would be very interesting to develop recommendations for AF management adapted to the Black population living in Africa.

## Conclusions

The prevalence of AF was 5.27%. Patients were younger and predominantly hypertensive. Patients presented to the hospital at an advanced stage of symptomatology, with dyspnea and palpitations being the main symptoms. The majority of patients were eligible for anticoagulant therapy, with VKAs being the most accessible. The low use of DOACs is related to availability, cost, or local guidelines. Stroke occurred in 11% of cases, highlighting the need to optimize preventive therapeutic strategies. Rate control was favored due to the inaccessibility of advanced rhythm control therapies. As a result, the frequency of restoration of sinus rhythm was relatively low. It is essential to focus efforts on the prevention of AF, especially through appropriate management of risk factors.
